# Endoscopic Ultrasound‐Guided Tissue Acquisition for Breast Cancer Liver Metastases Enables the Detection of Biomarkers Essential for Treatment

**DOI:** 10.1002/cnr2.70414

**Published:** 2025-12-05

**Authors:** Yuichi Takano, Naoki Tamai, Masataka Yamawaki, Jun Noda, Tetsushi Azami, Fumitaka Niiya, Naotaka Maruoka, Tatsuya Yamagami, Masatsugu Nagahama

**Affiliations:** ^1^ Division of Gastroenterology, Department of Internal Medicine Showa University Fujigaoka Hospital Yokohama Japan

**Keywords:** biomarker, breast cancer, endoscopic ultrasound‐guided tissue acquisition, estrogen receptor, human epidermal growth factor receptor‐2

## Abstract

**Background:**

Evaluating biomarkers, such as estrogen receptor (ER), progesterone receptor (PR), and human epidermal growth factor receptor‐2 (HER2), in pathological specimens is crucial for guiding breast cancer treatment. Since biomarker expression may differ between primary and metastatic lesions, biopsy of metastatic sites is recommended whenever feasible. The liver is a common metastatic site for breast cancer, and percutaneous biopsy has traditionally been the standard approach. Recently, endoscopic ultrasound‐guided tissue acquisition (EUS‐TA) has emerged as an alternative method for sampling focal liver lesions. However, the utility of EUS‐TA in assessing breast cancer biomarkers remains unclear.

**Aim:**

To evaluate the diagnostic performance of EUS‐TA, including its ability to assess biomarkers in liver metastases from breast cancer.

**Methods:**

This single‐center, retrospective observational study included patients who underwent EUS‐TA for breast cancer liver metastases between 2016 and 2023. Clinical characteristics and specimen adequacy were analyzed. A pathologist classified the obtained tissue samples into four categories: (A) insufficient for diagnosis, (B) diagnosis possible only at the cytology level, (C) histological evaluation possible, but additional immunostaining for biomarkers not feasible, and (D) histological evaluation and additional immunostaining for biomarkers feasible.

**Results:**

Fifteen cases were included, with a median patient age of 68 years (all female). The median liver lesion size was 20 mm (range: 8–50 mm). The lesions were located in the left lobe in 12 cases and the right lobe in 3 cases. A 22G needle was used in 14 cases, while a 25G needle was used in 1 case. Specimen adequacy was classified as follows: category A in 1 case (6.6%), B in 2 cases (13.3%), C in 0 cases (0%), and D in 12 cases (80%). Biomarker evaluation was feasible in the majority of cases. No procedure‐related adverse events were observed.

**Conclusion:**

EUS‐TA is a valuable method for obtaining tissue samples from breast cancer liver metastases, enabling biomarker assessment in most cases.

## 1 Introduction

In breast cancer treatment, evaluating biomarkers—estrogen receptor (ER), progesterone receptor (PR), and human epidermal growth factor receptor 2 (HER2)—is strongly recommended in clinical guidelines, as they serve as key predictors of treatment outcomes [1]. Endocrine therapy is effective for hormone receptor‐positive cases, while anti‐HER2 therapy benefits HER2‐positive cases [2, 3, 4, 5]. In contrast, treatment options for triple‐negative breast cancer, which lacks all three biomarkers, are limited, and prognosis remains poor [6].

Biomarker assessment is typically performed using immunohistochemistry (IHC) on tissue samples from the primary tumor. However, discrepancies in biomarker expression between primary and metastatic lesions have been reported [7, 8, 9, 10]. Therefore, whenever feasible, biopsies from metastatic sites should be obtained to reassess biomarker status.

The liver is a common metastatic site in breast cancer. Traditionally, percutaneous liver biopsy has been used for tissue sampling [11]. More recently, endoscopic ultrasound‐guided tissue acquisition (EUS‐TA) has emerged as a potential alternative [12]. EUS‐TA employs finer needles (19G–25G) compared to percutaneous biopsy (16G–20G), raising concerns about its adequacy for biomarker evaluation in breast cancer metastases. Whether EUS‐TA specimens are suitable for biomarker assessment in metastatic breast cancer remains unclear.

The aim of this study was to evaluate the diagnostic performance of EUS‐TA, including biomarker assessment (ER, PR, HER2), for breast cancer liver metastases.

## 2 Material and Methods

This single‐center, retrospective observational study was conducted at Showa University Fujigaoka Hospital and was approved by the institutional ethics committee of Showa University.

Clinical background and pathological diagnostic accuracy were assessed using medical records. Written informed consent was obtained from all patients involved in the study. The study was conducted in accordance with the principles of the Declaration of Helsinki.

### 2.1 Institutional Biopsy Strategy for Focal Liver Lesions

At our institution, EUS‐TA is the primary method for biopsy of focal liver lesions. However, percutaneous biopsy may be selected in cases where it is technically straightforward, such as for large lesions (> 5 cm) or lesions located on the liver surface, at the discretion of the operator. Conversely, percutaneous biopsy is also chosen when EUS‐TA is not feasible due to factors such as surgically altered anatomy, pharyngeal or esophageal strictures, or cardiorespiratory conditions that preclude sedation [13].

### 2.2 EUS‐TA Procedure

During EUS‐TA, analgesics and sedatives (pethidine hydrochloride [35 mg] or pentazocine [7.5–15 mg] combined with midazolam [1.5–4.0 mg]) were administered. The procedure was performed using a GF‐UCT260 endoscope (Olympus Medical Systems, Tokyo, Japan) and a UE‐ME1 or UE‐ME2 observation device (Olympus Medical Systems). The choice of puncture needle gauge (22G–25G) was at the operator's discretion. Each procedure involved 10–20 strokes with a suction pressure of 10–20 mL (negative pressure). If the obtained specimen contained excessive blood, the slow‐pull technique was applied. Rapid on‐site cytology (ROSE) was not performed.

Macroscopic on‐site evaluation (MOSE) was conducted by an endoscopist, and the procedure was terminated upon confirming the presence of white core tissue. The needles used included Expect SlimLine (Boston Scientific Japan, Tokyo, Japan) and SonoTip TopGain (Medico's Hirata, Tokyo, Japan). Expect SlimLine was a fine needle aspiration (FNA) needle, while SonoTip TopGain was a fine needle biopsy (FNB) needle. Contrast‐enhanced EUS was not performed in this study. All procedures were conducted by endosonographers with experience in performing more than 30 EUS‐TA cases.

### 2.3 Pathological Examination

Tissues obtained via EUS‐TA were fixed in 10% formalin, followed by histological diagnosis using hematoxylin and eosin (H&E) staining. IHC for biomarkers was performed whenever possible. After formalin fixation, the remaining liquid component was subjected to cytological examination using Papanicolaou staining.

Pathological specimens were assessed by a pathologist using a modified classification system from a previous study [14]:

- Category A: Insufficient for diagnosis (including cases where only necrotic tissue was obtained).
- Category B: Diagnosis possible only at the cytology level (tissue architecture not evaluable).
- Category C: Histological evaluation possible, but additional immunostaining for biomarkers not feasible.
- Category D: Histological evaluation and additional immunostaining for biomarkers feasible.
- ER positivity was defined as ≥ 10% of tumor cells staining positive in IHC. PR positivity was defined as ≥ 1% of tumor cells staining positive [15]. HER2 positivity was defined as a score of 3+ (complete circumferential membrane staining in ≥ 10% of tumor cells) [16].

### 2.4 Adverse Event Definition

Adverse events were defined according to the criteria established by the American Society for Gastrointestinal Endoscopy (ASGE) workshop [17].

## 3 Results

### 3.1 Case Selection

Between 2016 and 2023, EUS‐TA was performed on 18 cases of suspected breast cancer liver metastasis. Three cases were excluded due to a pathological diagnosis inconsistent with breast cancer metastasis (two cases of intrahepatic cholangiocarcinoma and one case of colorectal cancer liver metastasis).

Among the remaining cases, 12 were histologically confirmed as breast cancer liver metastases. In two cases, malignant cells were detected through cytology, and the clinical course further supported a diagnosis of breast cancer metastasis. In another case, no malignant findings were initially observed pathologically, but tumor growth over time led to a final diagnosis of breast cancer liver metastasis. Ultimately, 15 cases with a confirmed diagnosis of breast cancer liver metastasis were included in this study.

### 3.2 Clinical Background

The median age of patients was 68 years (range: 37–89 years), and all cases were female. The median size of liver lesions was 20 mm (range: 8–50 mm). Twelve cases had lesions in the left hepatic lobe: four in segment 2, six in segment 3, and two in segment 4. In contrast, three cases had lesions in the right hepatic lobe, with one in segment 6 and two in segment 8. None of the patients were taking oral antithrombotic drugs (Table 1).

**TABLE 1:** Clinical backgrounds of the cases.

	EUS‐TA for breast cancer liver metastasis (*N* = 15)
Age, median (range), years	68 (37–89)
Female, no. (%)	15 (100)
Lesion size, median (range), mm	20 (8–50)
Left lobe lesions, no. (%)	12 (80)
Segment 2, no	4
Segment 3, no	6
Segment 4, no	2
Right lobe lesions, no. (%)	3 (20)
Segment 6, no	1
Segment 8, no	2

### 3.3 EUS‐TA Procedure

EUS‐TA was performed via a transgastric approach in 12 cases and via a transduodenal approach in three cases (all from the duodenal bulb). The needles used were 22G FNB needles in 12 cases, 22G FNA needles in two cases, and a 25G FNA needle in one case. The median number of punctures was two. No procedure‐related adverse events were observed (Table 2).

**TABLE 2:** Details of EUS‐TA.

	EUS‐TA for breast cancer liver metastasis (*N* = 15)
Transduodenal puncture, no. (%)	3 (20)
Transgastric puncture, no. (%)	12 (80)
25 gauge FNA needle, no. (%)	1 (6.6)
22 gauge FNA needle, no. (%)	2 (13.3)
22 gauge FNB needle, no. (%)	12 (80)
Number of punctures, median (range)	2 (1–2)
Adverse events	0 (0)

### 3.4 Pathological Outcomes

**FIGURE 1:**
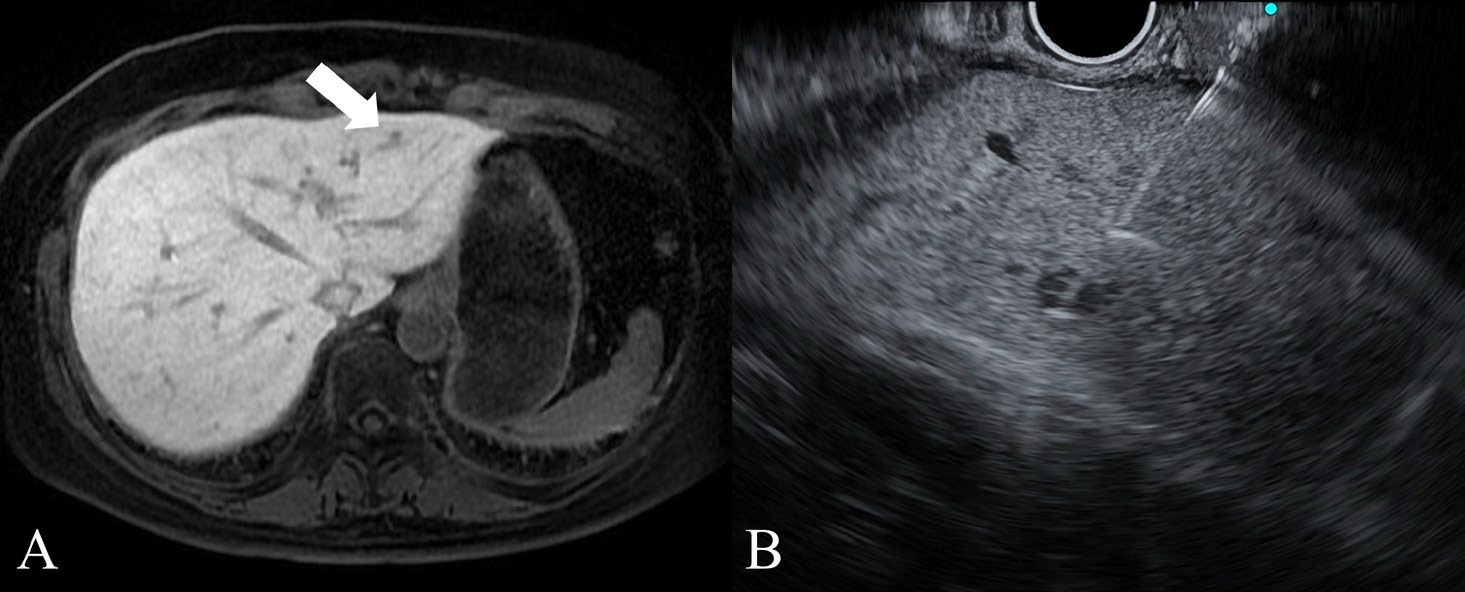
(A) Contrast‐enhanced magnetic resonance imaging revealed a small focal liver lesion (< 10 mm), located in segment 3 of the liver's lateral section (arrow). (B) EUS‐TA was performed to obtain a sample from the focal liver lesion.

**FIGURE 2:**
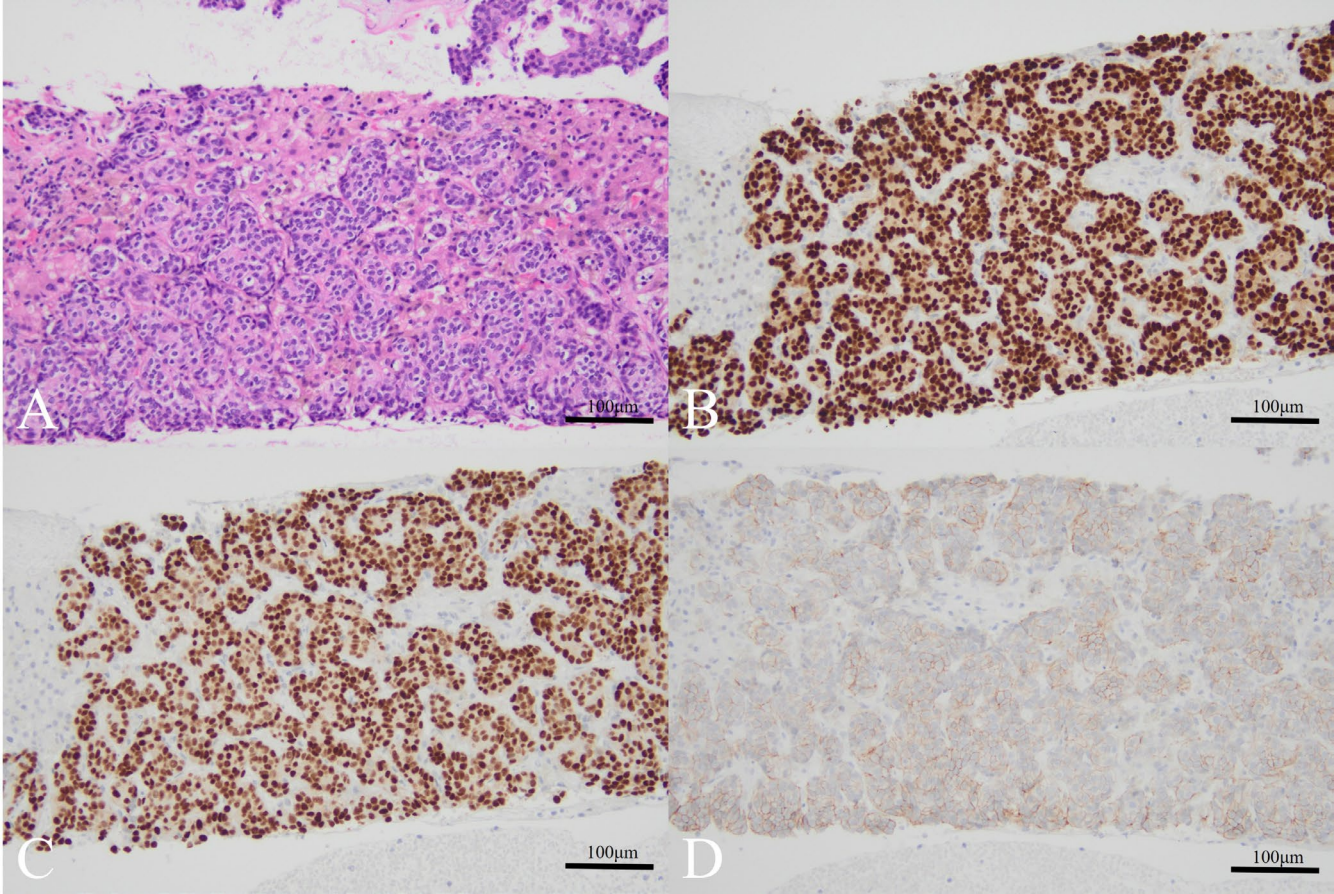
(A) Pathological examination identified atypical cells consistent with liver metastasis from breast cancer (H&E stain, ×20). (B) The tumor cells tested positive for the estrogen receptor (ER stain, ×20). (C) The tumor cells tested positive for the progesterone receptor (PR stain, ×20). (D) HER2 expression was evaluated as 2+ (HER2 stain, ×20).

The categorization results were as follows: Category A: 1 case (6.6%), Category B: 2 cases (13.3%), Category C: 0 cases (0%), Category D: 12 cases (80%). Biomarker evaluation was possible in 12 cases, with the following results: ER‐positive: 7 cases (46.6%), PR‐positive: 5 cases (33.3%), HER2‐positive: 3 cases (20%) (Figures 1 and 2) (Table 3).

**TABLE 3:** Pathological outcomes.

	EUS‐TA for breast cancer liver metastasis (*N* = 15)
Category classification	
Category A, no. (%)	1 (6.6)
Category B, no. (%)	2 (13.3)
Category C, no. (%)	0 (0)
Category D, no. (%)	12 (80)
ER positive cases, no. (%)	7 (46.6)
PR positive cases, no. (%)	5 (33.3)
HER2 positive cases, no. (%)	3 (20)
Diagnostic yield	
To detect malignant findings, (%)	93.3
To evaluate biomarkers (ER, PER, HER2), (%)	80

The diagnostic yield of EUS‐TA for detecting malignant findings in breast cancer liver metastases was 93.3% (14/15), while its yield for biomarker evaluation (ER, PR, HER2) was 80% (12/15).

The false‐negative case in which malignancy could not be diagnosed was performed using a 22‐gauge FNA needle. Among the three cases in which biomarker evaluation was unsuccessful, two involved the use of 22‐gauge FNA needles and one involved a 22‐gauge FNB needle.

## 4 Discussion

Evaluating biomarkers in breast cancer is essential, as it directly influences treatment strategies [1]. Previous studies have reported discrepancies in biomarker expression between primary and metastatic lesions, with rates ranging from 7%–30% for ER, 24%–40% for PR, and 10%–24% for HER2 [7, 8, 9]. In a large cohort of 542 patients with breast cancer liver metastases, discordance rates were reported as 21.0% for ER, 38.6% for PR, and 10.8% for HER2, underscoring that these findings are specific to liver metastases [10].

Consequently, clinical practice guidelines recommend performing biopsies on metastatic lesions to assess biomarkers [1]. Even if biomarkers are negative in the primary lesion, their presence in metastatic lesions may introduce new treatment options for patients.

The liver is a common site of breast cancer metastasis. Traditionally, percutaneous biopsies using 16G–20G needles have been performed for biomarker evaluation [11]. More recently, EUS‐TA has gained attention as an alternative biopsy method for focal liver lesions. EUS‐TA is a safe and effective technique, with reported diagnostic accuracy rates of 88–100% and an adverse event incidence of 0%–6% [18, 19, 20, 21, 22, 23, 24, 25, 26, 27, 28, 29, 30]. The most commonly used needle size is 22G, though 19G, 20G, and 25G needles may be selected based on individual cases. A comparison between EUS‐TA and percutaneous biopsy for focal liver lesions demonstrated no significant difference in diagnostic accuracy; however, the incidence of adverse events was significantly lower in the EUS‐TA group (2%) than in the percutaneous biopsy group (17%), with most adverse events consisting of procedure‐related pain [30].

Since EUS‐TA employs finer needles than percutaneous biopsy, the obtained samples tend to be smaller, raising concerns about the feasibility of biomarker evaluation in breast cancer liver metastases. In this study, among 15 cases with a final diagnosis of breast cancer liver metastasis, EUS‐TA enabled a malignant diagnosis in 14 cases (93.3%) and biomarker evaluation in 12 cases (80%). These findings suggest that EUS‐TA is a valuable biopsy method that allows for biomarker assessment in a majority of cases. Nevertheless, the occurrence of one false‐negative case and three cases in which biomarker assessment could not be performed should not be underestimated. Importantly, two of these three unsuccessful cases involved the use of FNA needles. Recent studies have demonstrated that FNB needles are more likely to yield specimens suitable for IHC evaluation [31]. Accordingly, when IHC is anticipated, the preferential use of FNB needles appears warranted. In all cases, the tumor size was 30 mm or larger, and the puncture route was transgastric. Although the number of diagnostic failures was too small to draw definitive conclusions, it is likely that the type of needle used (FNA needle), rather than tumor size or puncture route, contributed to the diagnostic failure.

In the early stages of EUS‐TA implementation, cytological examination was the primary diagnostic approach [18, 19, 20]. However, the recent widespread adoption of fine needle biopsy (FNB) needles has improved tissue yield, making it easier to obtain samples suitable for IHC [24, 28]. In the present study, FNB needles were used in 80% (12/15) of cases, which may have contributed to the high biomarker evaluation success rate. The results of this study suggest that EUS‐TA may serve not only as a salvage procedure when percutaneous biopsy is challenging but also as a first‐line biopsy method for breast cancer liver metastases. In particular, the lateral segments of the liver (S2, S3) are easily accessible for puncture using an echoendoscope; therefore, EUS‐TA may be actively considered.

Advantages of EUS‐TA of the liver include (1) Feasible in cases where percutaneous biopsy is challenging, such as those with Chilaiditi syndrome, ascites, or caudate lobe lesions, (2) the ability to perform the procedure under sedation, thereby enhancing patient comfort; and (3) Causes minimal pain compared to percutaneous biopsy [30]. Disadvantages include (1) Requires sedation, making it unsuitable for patients with severe heart or respiratory failure, (2) Requires technical expertise, with a longer learning curve, (3) Carries a risk of gastrointestinal injury, and (4) Cannot be performed in patients with pharyngeal or esophageal strictures.

Gastrointestinal perforation during EUS procedures is rare, with cervical esophageal and duodenal perforations reported in 0.03%–0.06% and 0.029%–0.86% of cases, respectively [32]. A narrative review indicates that the overall adverse event rate of EUS‐TA is 0%–2.5% [33]. In contrast, systematic reviews of percutaneous liver biopsy report major complications in 2.4% and minor complications in 9.5%, with bleeding as the predominant serious event and a small but non‐zero mortality risk [34]. Although direct comparisons are limited, adverse events appear less frequent with EUS‐TA, supporting its favorable safety profile. However, no cost‐effectiveness studies have specifically examined EUS‐TA versus percutaneous liver biopsy in the setting of breast cancer liver metastases, especially including long‐term outcomes and treatment decision‐driven costs, which should be addressed in future work. It should be noted that percutaneous biopsy offers certain advantages over EUS‐TA, as the procedure is simpler and more versatile, and does not require sedation, which may result in lower costs.

Our findings carry important clinical and translational implications. EUS‐TA demonstrates a favorable safety profile and provides reliable biomarker assessment, thereby positioning it as a valuable modality in the era of precision oncology.

The following were the limitations of this study: (1) a single‐center retrospective study, and the number of cases was relatively small; and (2) bias in case selection cannot be denied. In the future, it would be desirable to conduct clinical studies at multiple institutions with a large number of cases.

## 5 Conclusion

In this study, we evaluated the diagnostic performance of EUS‐TA in breast cancer liver metastases. The diagnostic yield of EUS‐TA for detecting malignant findings in breast cancer liver metastases was 93.3% (14/15), while its yield for biomarker evaluation (ER, PR, HER2) was 80% (12/15). EUS‐TA may serve not only as a salvage procedure when percutaneous biopsy is challenging but also as a first‐line biopsy method for breast cancer liver metastases.

## Author Contributions

Y.T. contributed to the conception and design of the report; acquisition, analysis, and interpretation of the data; and drafting of the final manuscript. N.T., M.Y., J.N., D.M., T.A., F.N., N.M., T.Y., and M.N. contributed to the analysis and interpretation of the data and drafting of the final manuscript. All the authors have read and approved the final manuscript.

## Funding

The authors have nothing to report.

## Conflicts of Interest

The authors declare no conflicts of interest.

## Data Availability

The authors have nothing to report.
